# Supplementary material for: The causal role of circulating inflammatory markers in osteoporosis: a bidirectional Mendelian randomized study
Source: Front Immunol. 2024 Jul 18;15:1412298. doi: 10.3389/fimmu.2024.1412298 (PMC11291241; doi:10.3389/fimmu.2024.1412298)
Supplement: Supplementary file 1 [file DataSheet_1.zip › supplementary material/CIM-OSTEOPOROSIS /GCST90274807.csv_leave_one_out.pdf]

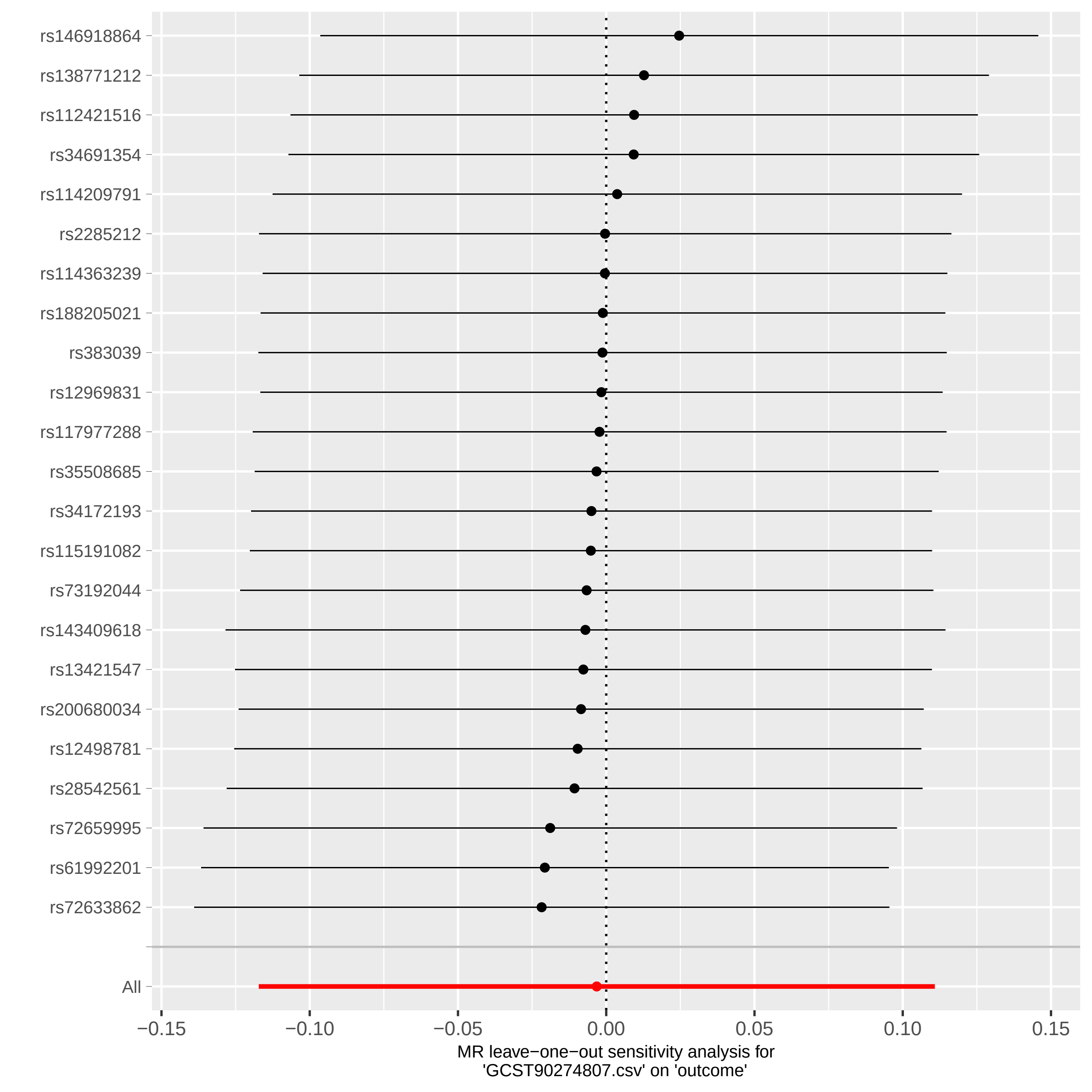

rs146918864
rs138771212
rs112421516
rs34691354
rs114209791
rs2285212
rs114363239
rs188205021
rs383039
rs12969831
rs117977288
rs35508685
rs34172193
rs115191082
rs73192044
rs143409618
rs13421547
rs200680034
rs12498781
rs28542561
rs72659995
rs61992201
rs72633862
All
−0.15
−0.10
−0.05
0.00
0.05
0.10
0.15
MR leave−one−out sensitivity analysis for
'GCST90274807.csv' on 'outcome'
